# From targeted therapy to a novel way: Immunogenic cell death in lung cancer

**DOI:** 10.3389/fmed.2022.1102550

**Published:** 2022-12-23

**Authors:** Jiawei Xu, Yiyi Xiong, Zhou Xu, Hongquan Xing, Lingyun Zhou, Xinyi Zhang

**Affiliations:** ^1^Department of Respiratory Diseases, The Second Affiliated Hospital of Nanchang University, Nanchang, China; ^2^The Second Clinical Medical College of Nanchang University, Nanchang, China; ^3^International Education College, Jiangxi University of Chinese Medicine, Nanchang, China

**Keywords:** immunogenic cell death, lung cancer, immunotherapy, damage-associated molecular patterns, genetic alteration

## Abstract

Lung cancer (LC) is one of the most incident malignancies and a leading cause of cancer mortality worldwide. Common tumorigenic drivers of LC mainly include genetic alterations of EGFR, ALK, KRAS, BRAF, ROS1, and MET. Small inhibitory molecules and antibodies selectively targeting these alterations or/and their downstream signaling pathways have been approved for treatment of LC. Unfortunately, following initial positive responses to these targeted therapies, a large number of patients show dismal prognosis due to the occurrence of resistance mechanisms, such as novel mutations of these genes and activation of alternative signaling pathways. Over the past decade, it has become clear that there is no possible cure for LC unless potent antitumor immune responses are induced by therapeutic intervention. Immunogenic cell death (ICD) is a newly emerged concept, a form of regulated cell death that is sufficient to activate adaptive immune responses against tumor cells. It transforms dying cancer cells into a therapeutic vaccine and stimulates long-lasting protective antitumor immunity. In this review, we discuss the key targetable genetic aberrations and the underlying mechanism of ICD in LC. Various agents inducing ICD are summarized and the possibility of harnessing ICD in LC immunotherapy is further explored.

## 1 Introduction

Over decades, lung cancer (LC) has remained one of the most frequently diagnosed cancers and ranks as the leading cause of cancer-related death in human globally (1). The 5-year survival rate is disappointing: only 19% of patients have survived overall, and most of them have suffered from a high risk of cancer relapse (2, 3). In the past decade, significant progress has been made in the treatment of LC. Researchers found several driver gene mutations of LC and deeply investigated molecular targeted therapy, which obviously improves the landscape of non-small cell lung carcinoma (NSCLC) treatment (4). Genetic alterations, such as mutations of epidermal growth factor receptor (EGFR), KRAS, MET, and rearrangements of ALK and ROS1, are the main contributors to LC tumorigenesis and progression (5), which dysregulate proliferation, apoptosis, migration, and invasion of cancer cells through various downstream signaling pathways. Targeting these abnormal genes or/and their downstream signaling has been used for treatment of LC. However, owing to the high heterogeneity of LC and acquired resistance to treatment, therapeutic efficacy of the targeted therapy is not guaranteed (6, 7).

Immunogenic cell death (ICD) is a novel form of regulated cell death which can evoke an adaptive immune response against cancer cells (8). Dying cancer cells can secrete damage-associated molecular patterns (DAMPs), mainly including high mobility group box 1 (HMGB1), calreticulin (CRT), adenosine triphosphate (ATP) and Type I interferon (Type I IFN). Recognized by the pattern recognition receptors (PRRs), the DAMPs enhance function of the antigen-presenting cells (APCs), activate T cells, increase the immunogenicity of tumor cells and ultimately trigger ICD (9). ICD can be triggered by many kinds of anti-cancer therapies, including chemotherapy, radiation, targeted drugs, photodynamic therapy (PDT) and immune checkpoints inhibitors (ICIs) (10–12). A shared characteristic of these various ICD is their ability to provoke endoplasmic reticulum (ER) stress and reactive oxygen species (ROS) generation. By restoring the immunogenicity of poor ICD triggers and stimulating DAMPs secretion, (ER) stress and ROS are believed to be indispensable for ICD (13, 14). Therefore, harnessing ICD to maintain the efficacy of anti-tumor therapies is crucial and challenging for LC treatment (15).

## 2 Genetic alterations affecting signaling pathways in lung cancer

**FIGURE 1:**
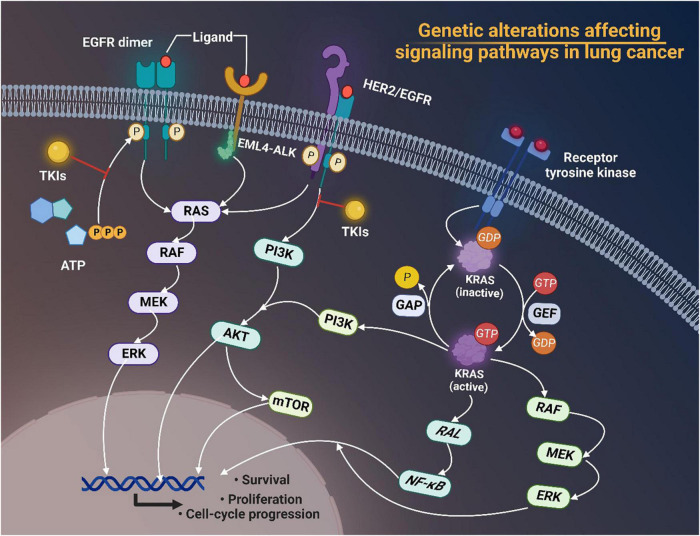
Genetic alterations of EGFR, ALK, and HER2 affecting signaling pathways in lung cancer. Tyrosine kinase inhibitors (TKIs) target receptor tyrosine kinases and prevent phosphorylation of the TK domain receptor of EGFR, thus inhibiting the activation of downstream signaling pathways such as the RAS/RAF/MEK/ERK pathway and the phosphatidylinositol 3-kinase (PI3K)-AKT pathway, thereby interfering with cell proliferation, differentiation, migration and survival. Activated KRAS proteins principally activate the downstream PI3K-AKT-mTOR signaling pathway that regulates cell proliferation and the RAS-RAF-MEK-ERK signaling pathway that regulates cell growth. Tumorigenic alterations contribute to EGFR overexpression, which ultimately increases the risk of cancer development by regulating related signaling pathways. EGFR, epidermal growth factor receptor; TKI, tyrosine kinase inhibitors; EML4-ALK, echinoderm microtubule associated protein like 4-activin-like kinase; HER2, human epidermal growth factor receptor 2; ATP, adenosine triphosphate; MEK, MAP kinase-ERK kinase; ERK, extracellular regulated protein kinases; PI3K, phosphoinositide 3-kinase; mTOR, mammalian target of rapamycin; GAP, growth-associated protein; GDP, guanosine diphosphate; GTP, guanosine triphosphate; GEF, Granule, Effervescent; RAL, Ras-like; NF-κB, nuclear factor-kappa B; RAF, rheumatoid arthritis factor; ERK, extracellular regulated protein kinases.

Mutations of EGFR, KRAS, BRAF, and MET, and rearrangements of ALK and ROS1 have aroused great interest in recent years. Plenty of studies have pointed out the significance of targeting tumor driver genes in cancer therapies nowadays due to their key roles in promoting cancer survival, proliferation and cell-cycle progression through modulating downstream signaling pathways in lung cancer (Figure 1).

### 2.1 EGFR mutations

**FIGURE 2:**
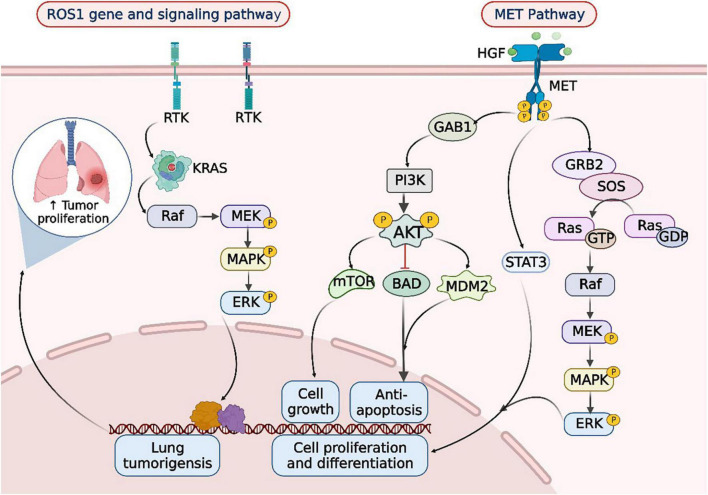
ROS1 rearrangements and MET mutations in the occurrence and development of lung cancer. When lung cancer is developed, the tyrosine kinase receptor (RTK) encoded by the ROS1 gene synergizes with oncogenic drivers such as KRAS to enhance MAPK-ERK signaling. Upon MET activation, PI3K associates with GAB1 and activates AKT/protein kinase. AKT inactivates the pro-apoptotic protein BCL-2 cell death antagonist (BAD) and triggers the E3 ubiquitin-protein ligase MDM2, thereby inhibiting apoptosis and promoting cell survival. In addition, AKT activates the mammalian target of rapamycin (mTOR) protein, promoting protein synthesis and cell growth, MET activation signals through the RAS-MAPK pathway as well. The nucleotide exchange protein Son of Sevenless (SOS) activates RAS upon binding to GRB2, which leads to activation of the v-Raf murine sarcoma viral oncogene homolog B1 (RAF) kinase, followed by stimulation of MAPK effector kinase (MEK) and resulting in MAPK activation. MAPK phosphorylates ERK, the ultimate effector of the cascade. The RAS-MAPK pathway is responsible for cell proliferation, cell motility, and cell cycle progression. Moreover, MET could relay signals to the activator of the transcription 3 (STAT3) pathway. STAT3 directly binds to MET, enabling STAT3 phosphorylation, which regulates cell transformation and invasion. RTK, receptor tyrosine kinases; Raf, rheumatoid arthritis factor; MEK, MAP kinase-ERK kinase; ERK, extracellular regulated protein kinases; HGF, hepatic growth factor; MET, mesenchymal to epithelial transition factor; GAB1, Grb2-associated binders 1; GAB2, Grb2-associated binders 2; PI3K, phosphoinositide-3 kinase; AKT, protein kinase B; mTOR, mammalian target of rapamycin; BAD, Bcl-xL/Bcl-2 associated death promoter; MDM2, murine double minute 2; SOS, son of sevenless; GTP, guanosine triphosphate; GDP, guanosine diphosphate; STAT3, signal transducer and activator of transcription 3.

For Asian patients with lung cancer, EGFR mutant NSCLC is the most prevalent subtype (16). Over the past decades, more and more studies have showed that EGFR mutation is a common driver of tumorigenesis, and lung cancer is no exception (17, 18). EGFR mutations include in-frame mutations or point mutations and insertions, which typically occurs in exon18-21, encoding a portion of the EGFR kinase domain. In-frame deletions in exon 19 and the L858R point mutation in exon 21 account for nearly 90% of EGFR mutations and confer high sensitivity to clinical target therapies (19). These mutations confer higher sensitivity to clinical target therapies due to increased affinity of TKIs to the ATP-binding pocket of mutant EGFR compared to its wild-type. However, insertion mutation and T790M point mutation in exon 20 are often resistant to TKI (20). EGFR mutations are responsible for activation of constitutive ligand-independent receptor and regulation of downstream signaling pathways, promoting cancer proliferation and cell survival (21). Regulated downstream signaling pathways include activation of RAS/RAF/MEK/ERK, phospholipase C (PLCγ) and phosphoinositide 3-kinase (PI3K)-AKT, but inhibition the p38/MAPK and JNK/STAT pathway (22) (Figure 2). Oncogenic alterations may result in EGFR overexpression as well, which eventually increases the cancer incidence risk *via* regulating related signaling pathway (23).

Mutant EGFR could inhibit apoptosis *via* inhibiting BH3 -domain proteins, such as pro-apoptotic BIM and BMF (24). Trever et al. proposed that the FAS/NF-κB pathway, which promotes tumor growth, could rescue EGFR mutant lung cancer cells from EGFR inhibition (25). Karachaliou et al. found that knockdown of Orphan receptor 1 (ROR1) could inhibit the growth of NCI-H1975 cells [harboring EGFR L858R and T790M mutations (24)] *via* the ROR1/MEK/ERK signaling pathway, indicating the potential of ROR1 as therapeutic target for EGFR positive lung adenocarcinoma (LUAD) (26). The study of Kinehara et al. proposed that the GPI-anchored protein semaphorin 7A (SEMA7A) is overexpressed *via* induction of mTOR signaling in LUAD. Mutant EGFR lung cancer could upregulate SEMA7A/ITBG1 axis, which normally activates ERK signaling and leads to apoptosis resistance (27). Zhang et al. reported that phosphohistidine phosphatase 1 (PHPT1), often overexpressed and caused poor survival in lung cancer patients, could activate ERK/MAPK pathway targeting F-box protein 32 (FBXO32) as E3 ubiquitin ligase in EGFR mutant lung cancer (28). Previous studies have revealed that betacellulin (BTC) could binds to members of the ErbB family and mediating cancer development. Chava et al. firstly proposed that BTC could suppress apoptosis and promote cancer growth in EGFR-mutant LUAD in a MAP kinase-dependent way (29). The F-box protein FBXL2, a potential therapeutic target for EGFR mutant LC, could suppress EGFR-driven NSCLC cell growth. Niu et al. showed that glucose-regulated protein 94 (Grp94) protects the stability of EGFR *via* blockage of FBXL2, thereby promoting EGFR mutant cell proliferation and anti-apoptosis (30).

Epidermal growth factor receptor-positive lung cancer could confer potent invasive ability. Tsai et al.’s study showed that EGFR-L858R mutant LUAD could activate CXCL12-CXCR4 axis to enhance metastasis (31). Feng et al. reached a similar conclusion in their study that EGFR 19 exon deletion can promote the expression of MMP-2 and MMP-9 by enhancing the CXCR4/CXCL12 signaling pathway, leading to higher proliferation, migration, and invasion abilities (32). Li et al.’s study found that the EGFR-mutant NSCLC related brain metastasis is associated with downregulation of WNT5A by E2F1 *via* ERK1/2 pathway (33). A recent study elucidated that Y-box Binding protein (YB-1), an important drug sensitivity modulator, could activate AKT signaling and epithelial-to-mesenchymal transition (EMT) *via* targeting major vault protein (MVP), especially in EGFR mutant LAUD (34).

Moreover, the crosstalk of EGFR with the tumor microenvironment (TME) could affect the immunity to cancer. High level of IL-6 is a biomarker in lung cancer patients, Cao et al. revealed that mutant EGFR could upregulate IL-6 *via* gp130/JAK signaling pathway targeting signal transducer and activator of transcription (STAT) 3, a known oncogenic protein (35). Patients with EGFR activation showed upregulated CD73/adenosine *via* EGFR-ERK signaling pathway, which contributes to the immune-inert environment for EGFR-mutant NSCLC (36–39). Chen et al. reported that activated EGFR NSCLC cells enhance ILT4 expression, which suppresses T cell proliferation and immunity and thereby leads to immune escape (40).

### 2.2 KRAS mutations

RAS is the most frequent mutant oncogene in cancer (41, 42). Among all kinds of isoforms, KRAS, which belongs to GFPase superfamily, accounts for 86% RAS-mutants (43). Research demonstrated that 20–40% of LUAD and 30% NSCLC have been observed with KRAS mutation, which is more prevalent in smokers (44). KRAS mutation could destruct the activation of GTPase, which would lead to the accumulation of KRAS under GTP binding condition and thus cause the activation of basic downstream pathways related with cellular life events, including MAPK, PI3K, and Ras-like (Ral) 2 guanine nucleotide exchange factor (RalGEF) and so on (45, 46).

KRAS mutations act as strong drivers for tumorigenesis by modulating multiple signaling pathways. It has been demonstrated that mutant RAS gene, could activate Raf–MEK–ERK phosphorylation cascade to enhance tumorigenesis (47). SIRT1, an oncogene or tumor suppressor, was found decreased by KRAS in a PI3K and MEK dependent way, which contributes to lung carcinogenesis (48). RASSF1A is believed to be a weak suppressor of human tumors. Transgenic mice with RASSF1A-defective background demonstrated that the loss of RASSF1A apparently enhances the RAS-driven lung cancer (49). EGFR palmitoylation has been shown to inhibit EGFR activity and alter downstream signaling in the KRAS mutant lung cancer. Blocking EGFR palmitoylation decreased PI3K signaling, negatively regulating lung carcinogenesis (50, 51). The ERBB/EGFR signaling pathway is also dysregulated in lung cancer. The activation of KRAS induces the phosphorylation of iRhom2, which induces excessive shedding of ERBB ligand and tumorigenesis (52). Escaping from oncogene surveillance is a vital part in tumorigenesis. RUNX3, which serves as a mediator of multiple tumor suppressor pathways, is inactivated in KRAS mutant lung cancer. KRAS-activated cells could develop into ADCs when Runx3-mediated tumor suppress signaling pathways are abrogated (53).

Accumulating evidence suggests that inflammation is an essential factor for tumor promotion (54). The JAK-STAT pathway is considered as a central player for inflammation mediated tumorigenesis and targeting this pathway in KRAS-driven LC has been proposed. KRAS mutant LC secrete pro-inflammatory cytokines which activate JAK1 and JAK2, thereby improving cell survival. Besides, deletion of STAT3 could enhance KRAS-driven lung cancer development (55). Previous studies have found that the abnormal activation of STAT3 in the development of KRAS mutant lung cancer, which will be attenuated under anti-IL-6 therapy, suggesting a tight association between IL-6/STAT3 signaling and inflammation in KRAS-activated tumorigenesis (56, 57). A study established a murine model and confirmed that this route is gender-specific, deletion of epithelial STAT3 in KRAS mutant LC female mice will decrease tumorigenesis, while the outcome is completely opposite in male mice (58). Later, their team showed that NF-κB is activated in KRAS-driven mouse model of LUAD (59). Bassères et al.’s work indicated that NF-κB is significant in KRAS-driven tumorigenesis, as the deficiency of p65/RelA profoundly impairs KRAS-driven lung tumorigenesis. Besides, inhibition of IKKβ expression suppressed NF-κB expression in KRAS-driven lung cells (60).

More and more studies have explained the underlying mechanism from various perspectives including proliferation, invasion and EMT. By upregulating DUSP6, a negative regulator of p-ERK, KRAS mutant lung cancer retrained the ERK1/2 mediated toxicity and promote cell proliferation (61). Wang et al. demonstrated that mutant KRAS could enhance the Cathepsin L/CUX1 axis, thereby promoting lung cancer invasion and migration (62). Hsu et al. firstly reported Yes-associated protein (YAP) in LUAD, their study stressed that YAP promote the brain metastasis of NSCLC cell lines H2030-BrM3 (KRASG12C mutation), especially by, targeting the downstream genes CTGF and CYR61 (63).

### 2.3 BRAF mutations

V-raf murine sarcoma viral oncogene homolog B (BRAF), which plays a vital part in cell proliferation, differentiation, and growth through mediating the MAPK pathway, belongs to the RAF family of serine/threonine protein kinase (64). Over 40 missense mutations have been discovered in human, while the most common BRAF mutation occurs in exon 15 is a thymidine to adenosine transversion at the level of T1799A, leading to the valine to glutamate substitution at codon 600 (V600E), which contribute to approximately half of the BRAF-mutant NSCLC (65, 66). This alteration could result in the activation of B-RAF kinase and constitutive MAPK/ERK cascade signal transduction, which leads to 500 folds BRAF activity compared with WT (67).

Mutant BRAF plays a positive key player in the tumorigenesis (68). Li et al. reported that ARHGEF19, one of Rho guanine nucleotide exchange factors (RhoGEFs), could interact with BRAF and promote MEK1/2 phosphorylation during the NSCLC formation (69). Tumor necrosis factor receptor (TNFR)–associated factors (TRAF) is a kinases modulator of TNFR family. Wang et al. firstly proposed the relationship between BRAF and TRAF1. The study explained that overexpressed TRAF1 could regulate BRAF/MAPK/ERK axis to promote NSCLC cells’ viability (70). C-RAF, which could promote adenoma initiation and growth, belongs to RAF family as well. Zanucco et al. reported that elimination of BRAF in oncogenic C-RAF expressed alveolar epithelial type II cells inactivates MAPK signal and lung Tumor growth (71). Upregulated terminal differentiation-induced non-coding RNA (TINCR) is associated with poor survival in NSCLC patients, Zhu et al. pointed out that TINCR could target BRAF and mediate downstream MAPK pathway to promote NSCLC tumorigenesis (72).

Abundant evidence indicates that inactivation of BRAF could cause cancer cell apoptosis, thus demonstrating the necessity of mutant BRAF in tumor cells (73). Lin et al. comprehensively evaluated the molecular determinants of BRAF mutant in lung cancer. Their study summarized that inhibition of MEK/ERK signaling targeting p61VE could suppress the cell escape from BRAFV600E oncogenic inhibitions in NSCLC. Besides, they found the MAPK pathway could mediate EGFR signaling and alleviate the dependence on BRAFV600E (74). Kotani et al. analyzed the role of MAPK signaling in BRAFV600E mutant lung cancer. The results showed that EGFR signaling could govern MEK/ERK pathway more strongly, however, the situation is not the same in BRAFV600E mutant lung cancer, which induce the receptor tyrosine kinases (RTKs) resistance problem (75). However, a previous study claimed that although BRAFV600E could initiate some benign tumors, lung cancer is seldomly induced. Trejo et al. suggested that co-mutation of PIK3CAH1047R and BRAFV600E could promote lung cancer progression, including transformation of tumor phenotype into malignant and accelerate tumor growth rate (76). The team of Mcmahon also demonstrated similar conclusion. Their research confirmed that the co-mutant BRAFV600E and PIK3CAH1047R in alveolar type 2 pneumocytes accelerate cell dedifferentiation (77). Tumor suppressor STK11 (LKB1) gene is frequently deficient in lung cancer. A study revealed that Lkb1 loss could promote tumorigenesis in BRAFV600E induced LUAD (78).

### 2.4 ALK rearrangements

ALK gene locates on the short arm of chromosome 2 (2p23), and belongs to the insulin receptor superfamily (79). As a tyrosine kinase receptor, ALK generally expresses in the brain and spinal cord during embryo genesis and dominantly decreased following maturation (79). The high correlation between ALK and tumorigenesis was firstly identified in 1994 as a fusion partner of nucleophosmin (NPM) in anaplastic large-cell lymphoma (80). Further research revealed underlying mechanism of NSCLC genesis with ALK translocation, as well (81, 82). ALK rearrangement presented potent oncogenic drivers in approximately 5–6% of NSCLC patients population characterized with young age, barely smoking and adenocarcinoma histology (83, 84).

The most common fusion partner of ALK is EML4 (echinoderm microtubule associated protein like 4) (85). Heat shock protein 90 (Hsp90) is a novel cancer therapy target owning to its positive role in controlling oncogenic signaling proteins. Normant et al. proposed that inhibition of Hsp90 could decrease EML4-ALK thereby inducing tumor regression in ALK-driven NSCLC (86). The crosstalk between Programmed cell death-ligand 1 (PD-L1) and its receptor programmed cell death-1 (PD-1) conferred profound strength in immunotherapy. A study examined the role of ALK rearrangement in affecting PD-L1 expression, their result showed that EML4-ALK fusion could decrease the PD-L1 expression through suppression of PI3K–AKT or MEK–ERK signaling pathway, which contributes to the immune escape in NSCLC (87). Shen et al. reported that EML4-ALK G1202R mutation could increase the invasion and migration ability of A549 cells. Besides, their research proved the EML4-ALK G1202R mutation could lead to EMT phenotype transformation in NSCLC cells by activating STAT3/Slug pathway (88).

### 2.5 ROS1 rearrangements

The ROS1 gene, which is located on chromosome 6 (6q22.1), is a widely known proto-oncogenic gene that belongs to the sevenless subfamily of tyrosine kinase insulin receptor. ROS1 rearrangements were primarily described in glioblastoma, the close correlation with NSCLC was discovered in 2012 (89). The ROS1 tyrosine kinase has been discovered to play a vital role in plenty of intracellular signaling pathways (90).

The (c-ros oncogene1) ROS1 rearrangements, which drive malignant transformation of NSCLC, affect approximately 0.7–1.7% NSCLC patients (91). The fusion partners include CD74, SLC34A2, FIG, TPM3, SLC12A2, CCDC6, and SDC4, while CD74-ROS1 fusion is the most prevalent phenotype in NSCLC (92, 93). Gou et al. demonstrated that CD74-ROS1 mutation could lead to EMT and enhance the NSCLC invasion and migration ability by upregulating Twist1 (94). Chromosomal rearrangement of the Solute Carrier Family 34 Member 2 (SLC34A2)-ROS1 fusion accounts for more than 14% of all ROS1 fusion in NSCLCs. Cai et al.’s study revealed that BA/F3 fusion NSCLC cells (harboring SLC34A2-ROS1) could activate ROS1-SHP2 signaling to elevate PD-L1 expression and mediate immunogenicity (95). Interestingly, another study supplemented that ROS1-fusion positive NSCLC cells could target MEK/ERK signaling pathway to upregulate PD-L1 expression significantly (96).

### 2.6 MET mutations

The proto-oncogene MET, located on chromosome 7q31, is one of the tyrosine kinase receptors. HGF is a common ligand of MET, upon their binding, MET will be dimerized and auto-phosphorylate, thus leading to the activation of activity of intracellular tyrosine kinase. Activation of MET could lead to the modulation of multiple downstream signaling pathways including RAS/RAF/ERK/MAPKA, PI3K/AKT/mTOR, Wnt/β-catenin, STAT and so on, which play vital roles in regulating tumor growth, progression and migration (97). Aberrant activation of MET signaling pathway may contribute to the tumorigenesis process of lung cancer.

About 3–5% of NSCLC patients have MET mutations, most of which are adenocarcinoma. Up to date, various mutations of MET have been identified, including MET amplification, MET point mutations, exon 14 skipping mutation, fusions and overexpression, all of which are oncogenic in lung cancer (98). The skipping of MET exon 14 mutation occurs in 3% LUAD and 13–22% sarcomatoid lung cancer (99). Studies revealed that the exon 14 skipping could lead to the lack of Y1003-Cbl, a ligand mediating c-MET degradation and ubiquitination, which subsequently prolong the activation of c-MET, downstream proliferation, and tumorigenesis (100). MET amplification is another major subtype of MET mutations which occur mainly in TKI-resistance lung cancer. Because single amplification of MET rarely contributes significantly to cancer development, co-mutant of MET and other cancer drivers, such as EGFR, appear more commonly (101, 102). A study indicated that only high amplification level of MET could display an oncogenic effect (103). However, there’s still a lack of research to fully elucidate the mechanism of MET-driven tumor development.

Recently, more and more target therapies are focusing on mutant genes to improve the prognosis for lung cancer. However, disappointedly, clinical outcomes are not positive as expected. Firstly, the development of drug resistance is another unavoidable theme in lung cancer. Jackman et al. primarily defined acquired resistance when patients receive target therapy over 6 months but the disease progression keeps evolving (104). Accumulating evidence has elucidated that, although the acquired resistance mechanism varies, the main reasons are from three aspects: mutant gene modification, phenotypic transformation and alternative signal pathway activation (105). EGFR TKIs are the most commonly used target drugs in EGFR mutant lung cancer patients. Previous studies have revealed that the EGFR T790M mutation, MET amplification, epithelial-to-mesenchymal transition (EMT), activation of the NF-κB pathway, and so on are common foundations of later developed EGFR TKIs resistance (105–108). These various factors could induce signaling pathway cross talk in lung cancer. Hepatocyte growth factor (HGF) could induce acquired resistance to TKIs by restoring the PI3K/Akt signaling pathway *via* phosphorylation of MET in EGFR mutant NSCLC (109). Activation of Hedgehog (Hh) pathway is a common feature of TKIs resistance. A study fully illustrated the role of Hh signaling in EGFR mutant lung cancer, their result showed that, through the induction of mesenchymal properties, Hh could mediate the resistance of EGFR inhibitors (110). Several clinical studies reported acquired resistance to targeted drugs, such as Osimertinib, a MET tyrosine kinase inhibitor (111). The advance in K-RAS targeted medicine faced huge challenge (112). On one hand, clinical trial of K-RAS targeting drugs revealed disappointing results. It was found that K-ras could activate an alternative pathway *via* geranylation with resistant farnesyl transferase inhibitors (113). Blumenschein et al. reported that the downstream MEK signaling pathway inhibitor failed to achieve significant positive effect on improving the survival of lung cancer patients (114). ALK- independent resistance occurs as well. Activating bypass signaling pathways compromising KIT amplification, MAPK, MET amplification, EGFR and BRAF V600E leads to the ALK TKIs resistance (115–117). Additionally, more drugs targeting other mutant genes are under research, which is not warranted to have a certain therapeutic effect. What’s more, the narrow therapeutic window is worrying. For example, the second-generation EGFR TKI afatinib displayed an apparent adverse effect in clinical trials (118). Besides, recurrence or metastasis of lung cancer occurs frequently. Previous research has elucidated that brain relapse, bone relapse, and relapse of other organs can seriously affect the prognosis process of lung cancer (119, 120).

Taken together, gene alteration is a common and significant phenomenon in tumorigenesis progression. Slight changes in these central cancer-driven genes can lead to dysregulate in downstream signal pathways, which dramatically affect lung cancer from multiple perspectives (Figure 2). However, the therapeutic effect is not always as positive as considered, clinical feedback suggested that the developed drug resistance is uncontrollable (5). Appropriate drug concentration is still in preclinical trials and prognostic recurrence/metastasis is beyond expectation. Therefore, a novel way of overcoming existing dilemma is urgently needed.

## 3 ICD in lung cancer cells

**FIGURE 3:**
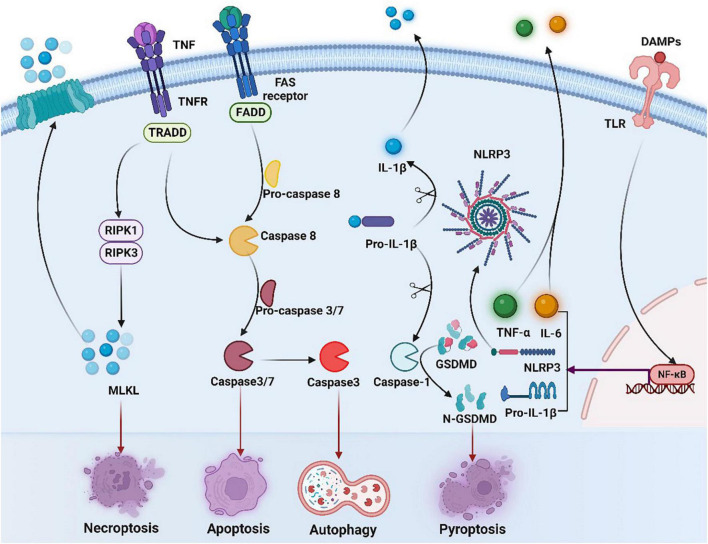
Overview of necroptosis, apoptosis, and pyroptosis signaling pathways. Binding of tumor necrosis factor (TNF) to its receptor (TNFR) activates RIPK3 *via* RIPK1, leading to the formation of necrosome, which activates mixed-lineage kinase-like (MLKL), contributing to necrosis and membrane permeation. Activation of caspase-8 induces exogenous apoptosis by triggering caspase-3 and caspase-7 activation. Meanwhile, caspase-3 induces autophagy. The occurrence of ICD in tumor cells is accompanied by the production of a series of signaling molecules in which released DAMPs can bind to pattern recognition receptors such as toll-like receptors (TLRs) on the surface of DC cells, activating NF-κB and inducing expression of NLRP3 and IL-1β/IL-18 precursors. NLRP3 recognizes various DAMPs and becomes oligomerized, contributing to activation of caspase-1 and production of mature IL-1β/IL-18. Activated caspase-1 cleaves gasdermin D (GSDMD) and releases the n-terminal (GSDMDNT) pore-forming fragments, thereby resulting in membrane permeation and pyroptosis. DAMPs, damage-associated molecular patterns; TLR, toll-like receptors; NF-κB, nuclear factor-kappa B; IL-6, interleukin-6; TNF, tumor necrosis factor, TNFR, tumor necrosis factor receptor; TNF-α, tumor necrosis factor alpha; NLRP3, NOD-like receptor thermal protein domain associated protein 3; IL-1β, interleukin-1 beta; GSDMD, gasdermin D; FADD, Fas-associating protein with a novel death domain; TRADD, TNF receptor 1 associated *via* death domain; RIPK1, receptor interacting serine/threonine kinase 1; RIPK3, receptor interacting serine/threonine kinase; MLKL, mixed lineage kinase domain-like protein.

Immunogenic cell death is a novel type of cell death which primes an adaptive immune response (Figure 3). Its main feature is that dying cells can secrete DAMPs, including HMGB1, CRT, ATP, and Type I IFN, which can enhance the antigen presentation capability of APCs, activate T cells, and enhance the immunogenicity of tumor cells, ultimately trigger the arise of ICD (9). In addition, induction of ER stress and ROS accumulation are indispensable components for ICD which increase the DAMPs (13, 14).

Apoptosis is one of the most researched forms of ICD. Apoptosis, mediated by the activity of caspases, is regulated by both endogenous and exogenous factors at the same time, and both of them rely on the activation of caspase-3 and caspase-7 (121). Yet, it has also emerged that non-apoptotic cell death can also be immunogenic, such as necrosis and pyroptosis. Necroptosis can be initiated firstly after activating the extended tumor necrosis factor alpha (TNF-α) receptor family on the surface of the cell, and transmitted in virtue of the serine/threonine kinases, RIP1 or RIP3 interact with receptors. Then dead cells release immunogenic DAMPs to greatly activate both innate and adaptive immune systems. Pyroptosis is a lytic pro-inflammatory modality of regulated cell death (RCD), which leads to the formation of plasma membrane pores with the help of members of the gasdermin protein family, particularly gasdermin D (GSDMD) (122). In addition, the anticancer immune response can be also triggered by autophagy, while autophagy was found to be related to the resistance of cancer cells to anti-cancer therapy (12).

### 3.1 Extra-cellular DAMP release

One of the main features of ICD is the release of molecular signals, which are usually called “DAMPs” (14). DAMPs can recognize specific receptors and attract adaptive immune cells like neutrophils, macrophages and dendritic cells (DCs). Then DAMPs promote activation and maturation of these immune cells, including dead cell removal, antigen uptake, processing, and presentation, and cytokine production (123). Some of the most widely researched ICD-linked DAMPs include HMGB1, CRT, ATP, and Type I IFN (124).

High mobility group box 1, which can trigger strongly inflammatory response when released from nucleus of dead cells, is an abundant nuclear non-histone chromatin-binding protein (125). HMGB1 binds to several receptors, such as Toll-like receptor 4 (TLR4), a type of receptor for advanced glycation end products (RAGE) to activate MAPKs and NF-κB in DCs, which are widely expressed in lungs (126–129). After CRT exposure, dead lung cancer cells will secrete HMGB1, which has a dual effect depending on whether it is extracellular or intracellular (14). Extracellular HMGB1 can facilitate the processing and presentation of antigens by DCs (128, 130), while intracellular HMGB1 can promote cancer cell growth and invasion, and resist therapy (129). Studies found that high level of HMGB1 is associated with poor prognosis in NSCLC (131). Moreover, Łagiedo et al. discovered that the levels of HMGB1 in NSCLC patients’ serum had a significant positive correlation with the size of the tumor (132).

Adenosine triphosphate is dependent on autophagy to be released from dying cancer cells in virtue of the active exocytosis of ATP-containing vesicles through pannexin channels (13, 133, 134). After being secreted out of cells and binding to purinergic receptor P2Y2 on the target cells, ATP will send a “find-me” signal to DCs and macrophages to promote DC maturation and macrophage expansion (135, 136). Moreover, ATP can mediate immune stimulation by activating the NLRP3 inflammasome and the subsequent secretion of interleukin 1 beta (IL-1β) (127, 137).

Calreticulin, a soluble protein in ER lumen, is exposed on the cell surface at a premortem stage and confers an “eat me” signal (122). After that, CRT interacts with the CD91 receptor in phagocytes to effectively engulf dead cells, thus providing abundant antigenic substance (13, 138). CRT also induces increased expression of endothelial cell adhesion molecules to promote infiltration of specific lymphocytes in the TME (139). Research has found that high CRT levels were shown to be in association with eIF2α phosphorylation in biopsies from NSCLC patients, which is independently relevant to better prognosis in NSCLC (140). Besides, in the treatment of lung cancer, CRT plays a similar role as HMGB1 so that it can be used to assess the extent of ICD induced by the treatment (141).

Type I IFNs, which can be driven by RNA or DNA species, are actively synthesized and activate other downstream genes including genes coding for chemokines to favor an immune response (142, 143). In RNA species, the receptor is endosomal TLR3, whereas the latter setting mainly works through cytosolic cyclic GMP-AMP synthase (CGAS) and its signal transducer stimulator of IFN response cGAMP interactor 1 (STING1) (144–146). Moreover, type I IFN can trigger macrophages to secrete pro-inflammatory mediators and inhibit the immunosuppressive functions of regulatory T cells (147, 148). Apart from these direct immunostimulatory functions, type I IFN can also elicit the synthesis of the CX-C motif chemokine ligand 10 (CXCL10) by tumor cells in ICD *via* an autocrine signaling loop (149).

### 3.2 ER stress

Immunogenic cell death can be divided into two modes according to its induction mechanism (13). Instead of inducing ROS and ER stress directly, type I ICD is stimulated by indirect signals. Quite the opposite, type II ICD targets the ER, inducing ER stress and immunogenic cell death (150). The process of ER stress activation is termed as unfolded protein response (UPR), featured with phosphorylation of eukaryotic translation initiation factor 2α (eIF2α) by Protein Kinase RNA-activated (PKR)-like ER Kinase (PERK) (151, 152). Several studies have shown that ER stress is the core of the occurrence of ICD (153). Moderate ER stress may be conducive to creating an immunosuppressive environment, while severe ER stress can stimulate immune response, as what happens in ICD (154). The more concentrated the ER stress is, the higher the immunogenicity of cell death is (155). ER stress is the main cellular mechanism for the cell surface exposure of CRT, which is closely linked to the phosphorylation of eIF2α. In Fucikova et al.’s study, they stated a subgroup of NSCLC associated with strong ER stress, which erupts in CRT expression and exposure (156). The high CRT driven by ER stress response has a positive prognostic value for NSCLC patients, however, its specific molecular mechanism remains to be further studied. ER stress also has an influence on levels of intracellular ATP by stimulating mitochondrial respiration, and cells can fill their bioenergy reserves in this way to restore cellular homeostasis (157).

## 4 Induction of ICD for lung cancer therapy

Immunogenic cell death can be caused by different types of stimulation and antitumor therapy, such as chemotherapy and radiation, some targeted drugs, oxygen-boosted PDT and ICIs. A lot of evidence shows that ICD can stimulate anticancer immune responses *in vivo*, and provide an opportunity to improve the cancer treatment and outcomes (158, 159). However, recently, only a few ICD inducers have been successfully translated into clinical practice. Here we elaborated on the role of ICD in the therapy of lung cancer.

### 4.1 Targeted therapy

Most anti-cancer drugs kill cancer cells in a non-immunogenic way. However, many studies demonstrated that ICD can be induced by different targeted agents. We will review these targeted drugs in detail to illustrate the important role of targeted therapy in the treatment of lung cancer (Table 1).

**TABLE 1:** Targeted therapies inducing immunogenic cell death (ICD) and their molecular targets in lung cancer treatment.

Drug	Molecular targets	Acquired mutations	Mechanisms	References
Crizotinib	ALK, ROS1	ALK, EGFR, KRAS, ROS1	Off-target effects	(124, 162, 210)
Ceritinib	ALK	ALK	Off-target effects	(124)
EnaV	AXL		Induce inflammatory response and the induction of a memory-like phenotype in cytotoxic T cells	(165)
Bemcentinib	AXL		Abrogate the transcription of autophagy-associated genes	(164)
BI2536	PLK1		Promote apoptosis and mitotic cell death, promote DC maturation and T-cell infiltration	(167)
Auranofin	Trx/TrxR	p53	Induce apoptosis and ferroptosis	(169)
Statins	RAS	KRAS	Induce severe ER stress	(170)
Biscoumarin OT52	STAT3	KRAS	Trigger cell cycle arrest and senescence, and multiple cellular stress mechanisms	(211)

EGFR, epidermal growth factor receptor; EnaV, enapotamab vedotin; DC, dendritic cell; Trx, thioredoxin; TrxR, thioredoxin reductase; ER, endoplasmic reticulum; STAT3, signal transducer and activator of transcription.

Crizotinib, a TKI used to treat NSCLC carrying activated ALK, ROS1, and MET, serves as an ICD stimulator *via* off-target effects (160). Drewry et al. have provided preclinical evidence that crizotinib can be expediently in combination with non-ICD inducing chemotherapeutics, as much with immune checkpoint blockade, to treat NSCLC in an effective way (161). One research has shown that the combination of cisplatin and high-dose crizotinib brings about an increase of PD-1 and PD-L1, and induces greatly ICD in NSCLC cells (12, 162). Hence, a sequential combination treatment including conventional chemotherapy together with crizotinib and immune checkpoint blockade may be effective for NSCLC. Besides, another ALK inhibitor ceritinib also has the function of targeting which can induce ICD in ALK-dependent NSCLC cell lines (124). For the past few years, the receptor tyrosine kinase AXL has been considered as a promising target for tumor treatment. The AXL signaling can promote a pro-survival pathway and reduce reactivation of the MAPK pathway to develop acquired resistance to EGFR TKI in NSCLC cells. And there is a positive correlation between AXL and autophagy (163, 164). Boshuizen et al. showed that an antibody–drug conjugate targeting AXL called enapotamab vedotin (EnaV) not only has direct tumor killing, but also induces inflammation and ICD of tumor cells in melanoma and lung cancer models (165). Lotsberg et al. reported that a small molecule inhibitor bemcentinib inhibits the transcription of autophagy-associated genes, releases DAMPs and then gives rise to ICD in NSCLC cells by targeted inhibition of the AXL signaling pathway (164). Erlotinib, a kind of TKI, can inhibit tumor development by inhibiting intracellular phosphorylation of EGFR-related tyrosine kinases, which commonly used in the treatment of NSCLC and pancreatic cancer. Studies have shown that targeting AXL in ER cells induces massive autophagic vacuolation before death in erlotinib-resistant cancer cells and triggers ICD (164). Hence, we suggest that ICD induction may have an unexpected effect on AXL-targeted NSCLC with drug-resistant EGFR mutations. Besides, PLK1 is a member of polo-like kinase family associated with cell division. PLK1 is overexpressed in NSCLC and it often predicts a poor prognosis (166). A selective PLK1 inhibitor BI2536 can act as an ICD inducer to cause apoptosis and alter the tumor immune microenvironment by promoting DC maturation and increasing T-cell infiltration (167).

In addition, several drugs widely used for other diseases have been found to have the ability to induce ICD in lung cancer cells. Auranofin (AF), a thioredoxin reductase 1 (TrxR) inhibitor, is known as an antirheumatic drug (168). TrxR is considered as a potential target in NSCLC on account of its high expression in NSCLC patients. A recent study indicated that AF can initiate release of DAMPs and DC maturation, then trigger apoptotic and ferroptotic cell death by targeting TrxR and launching ICD. It may provide new ideas for the treatment of NSCLC (169). Moreover, statins are one of the most frequently used drugs to treat hyperlipidemia. Statins can stimulate CD8 + T cells and provoke severe ER stress by inhibiting RAS prenylation in KRAS-mutant (KRASmut) lung tumor models, thereby leading to the ICD effects (170). Besides, coumarin is a kind of natural compounds with anti-inflammatory and anti-cancer function. Lee et al. found that biscoumarin OT52 strongly inhibited the proliferation of KRASmut NSCLC cells *via* ICD pathways (171). Mechanistically, biscoumarin OT52 suppresses STAT3 transactivation and expression of its target genes. Altogether, these drugs may become novel candidates in the future for more effective treatment of lung cancer. However, the specific signaling pathway in ICD inducing and other mechanisms still need to be explored in-depth.

### 4.2 Chemotherapy and radiation therapy

Chemotherapeutic drugs are considered to kill cancer cells selectively *via* direct cytotoxicity (12). A main mechanism of immunity stimulation by chemotherapy involves the induction of ICD (121). Unfortunately, only a little part of anti-cancer drugs can effectively trigger ICD (172). Some kinds of chemotherapeutic agents have been tried to modulate activity of DCs, such as cyclophosphamide, doxorubicin, oxaliplatin and anthracyclines, which can make tumor antigens be vaccines to the immune system and induce ICD, consequently provoking robust adaptive immune response (173–175). Chemotherapy with a combination regimen of oxaliplatin with cyclophosphamide is approved in clinical practice for lung cancer. Previous studies found that in a lung mouse cancer model, this regimen can foster CD8+ T cell infiltration and increase TLR4+ DCs in tumor tissues, and further increase tumor sensitivity to immune checkpoint therapy (176). But the research by Fileswasser et al. has shown that oxaliplatin does not induce ICD in NSCLC cells (4). Pemetrexed, a multi-targeting antifolate antagonist which is established as the main chemotherapy drug for the first-line treatment of advanced non-squamous NSCLC (NSq-NSCLC) and mesothelioma, has also been shown to induce ICD and to increase of immune-regulatory genes (177, 178). Liu et al. found that crizotinib, a kind of drugs used to treat NSCLC patients which carries activated ALK/ROS1, is an efficient ICD stimulator *via* off-target effects (162). Wang et al.’s results show that trametinib also has the ability to induce ICD by sensitizing lung cancer cells to endoplasmic reticulum stress and triggering the release of DAMPs, and can be effective in treating KRAS-mutant LUAD when used in combination with interleukin-12 (IL-12) (179). A study by Gao et al. showed that, DOC, a kind of tubulin stabilizer belonging to the taxane family, can induce DAMPs and significantly upregulated release of HMGB1 in human NSCLC cell line (180). In addition, the results of Furuwaka et al. suggest that osimertinib induced NSCLC tumor cell death may lead to exposure and release of CRT to induce ICD, and then improve the anti-tumor immunity (181).

As a topical treatment approach, radiation therapy (RT) is widely used in clinical cancer treatment. RT can induce ICD, which promotes DCs activation and the presentation of tumor antigen to prime CD8+ T cells (182). The CD8+ T cells then enter the unirradiated tumor area and attack cancer cells (14, 183). RT and many traditional chemotherapeutic agents give rise to DNA damage and multiform cell death ultimately (184). In various preclinical settings, similar to chemotherapy, induction of ICD by RT has been shown associated with increased sensitivity to immune checkpoint blockade (144, 185), and many clinical trials have proven that (186). In the same way, ablative RT can induce necroptosis in NSCLC and mediate HMGB1-driven immunological response (187).

### 4.3 PDT

Photodynamic therapy is able to kill cancer cells by manipulating photosensitizers and generating reactive ROS, which triggers ER stress and induces the anti-tumor immunity to eliminate residual or metastatic tumors effectively and selectively (155, 171, 188). After accumulating selectively in the tumor area, the photosensitizer (PS) is activated by illumination with visible light of appropriate wavelength, and then illuminated by red light (690 nm), which can induce local ICD at the tumor sites and strong anti-tumor immunity (10, 189). In recent years, the concept of PDT has been actively pursued. The binding of near-infrared PS to antibodies or nanocarriers improves the efficiency of PDT (190). One typical PS shown to induce ICD is hypericin, which is an anthraquinone derivative of natural origin with specific ER localization (155, 171). The other promising non-porphyrin PS is benzophenazine, OR141, which also has specific location in the ER (191). OR141 induces cell death mainly *via* the mammalian target of rapamycin signaling pathway and by inhibition of proteasomal deubiquitinases, leading to ER stress (192). One study reveals that in a prophylactic tumor vaccination model using PDT-treated TC1 lung cancer cells, redaporfin acts as an ICD inducer that can trigger eIF2a phosphorylation, DAMPs release and inhibit tumor growth (193). ICD can also be induced by PDT based on 8-methoxypsoralen (8- MOP) (194). But it is worth noting that it doesn’t need oxygen but intercalates into DNA and forms cross-links with one or two DNA strands under UVA irradiation (195). Furthermore, it has been shown that photofrin-based PDT of Lewis lung carcinoma cells induced release of HSPs, and surface exposure of CRT *in vitro* and *in vivo* in an hour after PDT, as well as an increase of HMGB1 (196). These data also indicate that photofrin is a potential inducer of ICD.

### 4.4 ICIs

The antitumor effect of ICIs works by interfering with immune tolerance (178). The most clinically common immune checkpoints include: PD-1/PD-L1, cytotoxic T lymphocyte-associated antigen-4 (CTLA-4), indoleamine 2.3-dioxygenase (IDO), and CD47 (197, 198). ICIs have established a new model of lung cancer treatment and improved patients’ survival benefits (199, 200). It also has revolutionized the prognosis of multiple lung cancers, especially NSCLC, which have a high sensitivity to the immunotherapy against PD-1 (201). The combination therapy of platinum, PEM and ICIs has been proposed as a standard first-line treatment for advanced LUAD (202, 203). Also, numerous studies have shown platinum-based combination chemotherapy and combination ICIs, like PD-1 or for its ligand PD-L1, can markedly prolong survival in patients with stage III unresectable NSCLC (204–207). In clinical application, the combination of ICIs and chemotherapy can improve the efficacy of anti-tumor therapy, this may be because chemotherapy drugs increase tumor sensitivity to ICIs (208). Pemetrexed and ICIs targeting PD-1/PD-L1 are applied widely for the treatment of advanced NSq-NSCLC (178). Moreover, lurbinectedin is a kind of DNA-binding inhibitors of transcription, which is efficient at inducing ICD (209). The combination therapy of lurbinectedin and ICIs targeting PD-1/PD-L1 is supposed to be a salvage therapy for relapsed SCLC be over the years (172).

## 5 Conclusions and perspectives

Worldwide, lung cancer is one of the most common cancers and the leading cause of cancer-related deaths. For decades, researchers are exploring the pathogenesis of lung cancer and trying to find more effective treatments, such as by finding oncogenic driver gene mutations to improve targeted therapy. However, it is disappointing that clinical results have not been as positive as we expected. In recent years, ICD was noticed for evoking adaptive immune response of cancer cells. ICD can be induced by a variety of anticancer therapies, including chemotherapy, radiotherapy, targeted drugs, PDT and ICIs, etc. And it is becoming increasingly evident that ICD may offer a new idea in the anti-cancer therapeutic approaches in the future, especially for lung cancer.

In summary, it is a breakthrough to harness ICD to elevate the immunogenicity of tumor cells to maintain the efficacy of anti-tumor therapies for lung cancer. ICD induction is a promising area to explore and the mechanism of function and regulatory networks of ICD deserve further investigation. Finally, due to the limitations of current study, there are still many unanswered questions, such as whether ICD is associated with ferroptosis or cuproptosis, whether ICD is associated with anti-angiogenic drugs, and so on.

## Author contributions

XZ: conceptualization and funding acquisition. JX and YX: writing – original draft preparation. ZX and HX: adapt the text and figures. XZ and LZ: writing – review and editing and project administration. All authors have read and agreed to the published version of the manuscript.
